# Glycosylation—The Most Diverse Post-Translational Modification

**DOI:** 10.3390/biom12091313

**Published:** 2022-09-17

**Authors:** Erika Staudacher, Els J. M. Van Damme, Guy Smagghe

**Affiliations:** 1 Department of Chemistry, University of Natural Resources and Life Sciences, 1190 Vienna, Austria; 2 Department of Biotechnology, Faculty of Bioscience Engineering, Ghent University, Proeftuinstraat 86, 9000 Ghent, Belgium; 3 Department of Plants and Crops, Ghent University, 9000 Ghent, Belgium

This article is part of the Special Issue Glycosylation—The Most Diverse Post-Translational Modification.

Glycosylation plays an important role in several types of biological and biochemical recognition processes, ranging from fertilisation and development to pathological events, such as infection, allergy, inflammation or cancer. Analyses of carbohydrate-based relations (host finding, recognition and invasion) between parasites and their hosts or intermediate hosts are attracting increased interest due to the implications in diagnostics, vaccine development, novel therapies and immune responses. The impact of glycosylation on the mobility of pathogens between species is of particular interest.

This Special Issue aims to highlight aspects of protein glycosylation in all phyla. The collection presented here includes four original research papers and three review articles covering glycosylation aspects from bacteria, plants, molluscs and humans. The broad spectrum of contributions illustrates the importance of the topic. Methodological aspects of glycan analytics are discussed, in addition to surprising modifications of glycans or medical aspects of glycans in diagnosis and therapy.

Are mosses plants? Or are they somewhere located between algae and plants? Stenitzer et al. [1] find, in their highly advanced mass spectrometry studies, an amazing amount of methylated *N*-glycan structures in various moss species. *O*-Methylation of oligosaccharides has been found in algae, but not in plants. However, the mosses display typical structural features seen in vascular plants. Depending on the species, protein-bound *N*-glycans are very heterogeneous; some are closer to plants, while others are closer to algae. 

Technical innovations have led to improvements in *N*-glycosylation analyses in recent years. The study of Helm et al. [2] uses today’s facilities to investigate the *N*-glycome of the human brain. In a technically complex study, typical structures of the brain were first bio-synthesised and then compared with 68 real *N*-glycan structures, with particular focus on the determination of isomers. In addition to complete coverage of the brain glycome, the innovative methodical workflow will allow faster and more reliable analysis of other tissues. 

Biosynthesis of *O*- and *N*-glycans requires specific glycosyltransferases. In the article of Tomek et al. [3] a new fucosyltransferase family encoded in Bacteroidetes protein *O*-glycosylation genetic loci is described. The enzymes of three different Gram-negative bacteria (*Tannerella forsythia*, *Bacteroides fragilis* and *Pedobacter heparinus*) were identified and characterised, all of which show a broad acceptor specificity spectrum, making them promising candidates for application in synthetic glycobiology. 

Pyruvylation (pyruvate–ketal modification) is a rare modification of glycans located in certain microorganisms. Hager-Mair et al. [4] present a method used to study the enzyme activity of a ketalpyruvyltransferase from *Paenibacillus alvei*. The new assay is based on the colorimetric detection of phosphate release during pyruvyltransfer from phosphoenolpyruvate onto the acceptor via complexation with Malachite Green and molybdate. This assay improves the analysis of this kind of enzymes; several synthetic acceptors have been found to be inadequate. 

Banerjee et al. [5] summarise the current state of treatments for breast cancer and introduce a new treatment option. Tunicamycin quantitatively inhibits in vitro and in vivo angiogenesis of multiple cancer subtypes in preclinical mouse models, blocking a specific step of the protein *N*-glycosylation pathway in the endoplasmic reticulum. This treatment inhibited breast tumour progression. Thus, tunicamycin could be used in glycotherapy against breast cancer in the future. 

Hirano and Furukawa [6] focus on a specific disaccharide linked to *O*- and *N*-glycans: the LacdiNAc (GalNAcβ1,4GlcNAc) group. The expression of this group is very tissue specific, and it is closely associated with tumour progression or regression. Although the biological role of this group in cancer cells is not yet fully understood, it is clear that LacdiNAc influences the development of breast cancer cells from normal to malignant phenotypes. Because of its association with tumour development, LacdiNAc is a potentially suitable tumour marker for some types of cancer. 

Staudacher’s review [7] summarises the current state of knowledge on *N*-glycosylation in molluscs. So far, this large phylum has been studied only superficially. The few studies available to date show that molluscs have an enormously wide range of glycan structures, and further modifications are linked to their sugars. In this review, the analysis of structures and studies on the corresponding enzymes, the involvement of glycans in the life cycle of parasites using snail intermediate hosts and potential medical applications of molluscan glycans are covered. 

This Special Issue contains a selection of highlights from the field of glycobiology. Methodological innovations, structural aspects, glycosyltransferases and medical aspects are described. This small selection of articles demonstrates the broad scope and potential of this research area. Glycans have an enormous biological, diagnostic and therapeutic impact. Glycosylation and its challenging structural and functional analysis will be subject to further investigation in the future.

## References

[1] Stenitzer D., Mócsai R., Zechmeister H., Reski R., Decker E.L., Altmann F. (2022). *O*-methylated N-glycans Distinguish Mosses from Vascular Plants. Biomolecules.

[2] Helm J., Hirtler L., Altmann F. (2022). Towards Mapping of the Human Brain N-Glycome with Standardized Graphitic Carbon Chromatography. Biomolecules.

[3] Tomek M.B., Janesch B., Braun M.L., Taschner M., Figl R., Grünwald-Gruber C., Coyne M.J., Blaukopf M., Altmann F., Kosma P. (2021). A Combination of Structural, Genetic, Phenotypic and Enzymatic Analyses Reveals the Importance of a Predicted Fucosyltransferase to Protein *O*-Glycosylation in the Bacteroidetes. Biomolecules.

[4] Hager-Mair F.F., Stefanović C., Lim C., Webhofer K., Krauter S., Blaukopf M., Ludwig R., Kosma P., Schäffer C. (2021). Assaying *Paenibacillus alvei* CsaB-Catalysed Ketalpyruvyltransfer to Saccharides by Measurement of Phosphate Release. Biomolecules.

[5] Banerjee D.K., Lebrón A.S., Baksi K. (2022). Glycotherapy: A New Paradigm in Breast Cancer Research. Biomolecules.

[6] Hirano K., Furukawa K. (2022). Biosynthesis and Biological Significances of LacdiNAc Group on *N*- and *O*-Glycans in Human Cancer Cells. Biomolecules.

[7] Staudacher E. (2021). Mollusc N-glycosylation: Structures, Functions and Perspectives. Biomolecules.

